# Development of a Risk Prediction Model for New Episodes of Atrial Fibrillation in Medical-Surgical Critically Ill Patients Using the AmsterdamUMCdb

**DOI:** 10.3389/fcvm.2022.897709

**Published:** 2022-05-13

**Authors:** Sandra Ortega-Martorell, Mark Pieroni, Brian W. Johnston, Ivan Olier, Ingeborg D. Welters

**Affiliations:** ^1^School of Computer Science and Mathematics, Liverpool John Moores University, Liverpool, United Kingdom; ^2^Liverpool Centre for Cardiovascular Science, Liverpool, United Kingdom; ^3^Institute of Life Course and Medical Sciences, University of Liverpool, Liverpool, United Kingdom; ^4^Liverpool University Hospitals NHS Foundation Trust, Liverpool, United Kingdom

**Keywords:** atrial fibrillation, critically ill patients, risk prediction, AmsterdamUMCdb, ICU

## Abstract

The occurrence of atrial fibrillation (AF) represents clinical deterioration in acutely unwell patients and leads to increased morbidity and mortality. Prediction of the development of AF allows early intervention. Using the AmsterdamUMCdb, clinically relevant variables from patients admitted in sinus rhythm were extracted over the full duration of the ICU stay or until the first recorded AF episode occurred. Multiple logistic regression was performed to identify risk factors for AF. Input variables were automatically selected by a sequential forward search algorithm using cross-validation. We developed three different models: For the overall cohort, for ventilated patients and non-ventilated patients. 16,144 out of 23,106 admissions met the inclusion criteria. 2,374 (12.8%) patients had at least one AF episode during their ICU stay. Univariate analysis revealed that a higher percentage of AF patients were older than 70 years (60% versus 32%) and died in ICU (23.1% versus 7.1%) compared to non-AF patients. Multivariate analysis revealed age to be the dominant risk factor for developing AF with doubling of age leading to a 10-fold increased risk. Our logistic regression models showed excellent performance with AUC.ROC > 0.82 and > 0.91 in ventilated and non-ventilated cohorts, respectively. Increasing age was the dominant risk factor for the development of AF in both ventilated and non-ventilated critically ill patients. In non-ventilated patients, risk for development of AF was significantly higher than in ventilated patients. Further research is warranted to identify the role of ventilatory settings on risk for AF in critical illness and to optimise predictive models.

## Introduction

Atrial Fibrillation (AF) is the commonest arrhythmia worldwide and increases the risk of stroke and heart failure (1). AF is characterised by irregular atrial electric activity and ventricular response. In the general population diabetes, high blood pressure and coronary artery disease are the main risk factors. AF is also common after major surgery and in patients suffering from acute severe illness, in particular infection (2). Up to 44% of all patients admitted to intensive care units suffer from incident AF (3–6). Many of these episodes occur in patients without a history of AF. The occurrence of AF in patients admitted with sinus rhythm represents a clinical deterioration in acutely unwell patients and leads to increased morbidity and mortality (7). The onset of AF is often associated with haemodynamic instability and usually requires treatment either with anti-arrhythmic drugs or electric cardioversion to control heart rate and rhythm.

Large repositories of Electronic Health Records have been used to develop risk prediction for AF in the general population using machine learning algorithms. A recent risk prediction model, developed for the general population, the CHARGE-AF (The Cohorts for Heart and Aging Research in Genomic Epidemiology AF) score, predicts an individual’s 5-year risk of new AF using clinical variables including age, ethnicity, height, weight, blood pressure, medication and comorbidities (8). Due to the long period covered and the differences in patient population, such models are not suitable for the prediction of AF in acute illness. Previous research on large datasets to explore incidence, risk factors and outcome of AF in acutely unwell patients focused on septic patients and relied on United States databases (9, 10).

In addition, risk factors for developing AF in the general population compared to critically ill patients may vary substantially. Traditional risk factors associated with AF in the community include structural and valvular heart disease, neither of which is clearly related to AF in critical illness (11). In addition, acute factors, rather than pre-existing cardiovascular comorbid conditions, are thought to be associated with increased risk for newly diagnosed AF during critical illness (9). In particular, invasive ventilation or the use of vasoactive drugs and inotropes may trigger episodes of AF in the critical care setting (11) but has no relevance in ambulatory care.

Over the last decade, the use of various modelling techniques for AF has grown exponentially (12) and includes detection as well as prediction of AF. Existing models of risk prediction for AF are based on specific cardiac cohorts (12) and are not easily transferable to critical care, despite the numerous reviews (11, 13–15) which describe the risk factors for AF in sepsis and critical illness. As a consequence, the identification of subsets of critically ill patients at risk of developing AF before its clinical manifestation requires improvement. To date the availability of sophisticated risk prediction models in critical care is limited. The few existing models focus on septic patients only (16) and do not include the large proportion of critically ill patients with non-infectious pathologies. Advanced models for prediction of AF during critical illness, but before its clinical onset, would allow early interventions with a view to preventing serious AF-associated complications, such as haemodynamic instability, stroke and thromboembolic events.

To date models focusing on prediction and detection of AF are mainly based on data from the Medical Information Mart for Intensive Care (MIMIC) III database, which comprises data obtained in a single large tertiary care centre in the United States. So far, European databases have not been used to identify risk factors or to construct prediction models for AF in critical illness. The Amsterdam University Medical Centers Database (AmsterdamUMCdb), endorsed by the European Society of Intensive Care Medicine (ESICM), is the first freely accessible European intensive care unit (ICU) database (17).

Here we present a logistic regression model for the prediction of AF in critical illness using the AmsterdamUMCdb database (18). In addition to static variables, we include time series of vital signs, blood results and ventilatory settings in septic and non-septic patients in this model. We develop different models to predict the first occurrence of AF in patients admitted to critical care in sinus rhythm and to identify factors associated with the occurrence of AF. Furthermore we differentiate between ventilated and non-ventilated patients to account for mechanical ventilation as an established risk factor for development of AF in critical care.

## Materials and Methods

### Data

We used data from the AmsterdamUMCdb, a freely available database, accessible after completing the mandatory training and guaranteeing the involvement of a practising intensivist in the research team to provide domain expertise. The database contains data from a 32-bed mixed surgical-medical academic ICU and a 12-bed high-dependency unit (medium care unit) (18). For patients who developed AF after ICU admission, the timestamp of the first episode of AF, as documented in the database, was used, while for non-AF patients the endpoint was the end of their ICU stay. Variables were extracted until 1 h before the first recorded AF episode for AF patients, whereas for non-AF patients data were analysed for the whole ICU stay. The interval of 1 h between the last data set included and the onset of AF was deliberately chosen because if applied in clinical practice, such a time frame would allow interventions to prevent the onset of the AF episode to be initiated. Variables included demographic data coded in classes (e.g., age, gender, weight and height), vital signs coded continuously (e.g., heart rate, breath rate, temperature, systolic blood pressure, and oxygen saturation), blood results and variables describing the level of respiratory support, such as FiO2 (Table 1).

**TABLE 1:** Demographics, vital signs, and routine prognostic scores used for modelling.

	AF (*N* = 2374)	Non-AF (*N* = 16144)	P-value
Location			<0.001
MC	150 (6.4%)	3308 (20.5%)	
IC	1837 (78.0%)	11797 (73.3%)	
IC&MC	369 (15.7%)	998 (6.2%)	
Urgency [yes]	826 (34.8%)	4382 (27.1%)	<0.001
Admission year group			0.098
2003–2009	1355 (57.1%)	9504 (58.9%)	
2010–2016	1019 (42.9%)	6640 (41.1%)	
ICU mortality	548 (23.1%)	1151 (7.1%)	<0.001
Gender [male]	861 (36.3%)	5556 (34.4%)	0.077
Age group (years)			<0.001
18–39	52 (2.2%)	2001 (12.4%)	
40–49	71 (3.0%)	1721 (10.7%)	
50–59	239 (10.1%)	2973 (18.4%)	
60–69	586 (24.7%)	4354 (27.0%)	
70–79	911 (38.4%)	3874 (24.0%)	
80+	515 (21.7%)	1221 (7.6%)	
Weight group (kg)			0.437
59–	204 (8.8%)	1214 (7.8%)	
60–69	354 (15.3%)	2513 (16.1%)	
70–79	608 (26.3%)	4309 (27.6%)	
80–89	613 (26.5%)	3980 (25.5%)	
90–99	316 (13.7%)	2134 (13.7%)	
100–109	120 (5.2%)	818 (5.2%)	
110+	98 (4.2%)	627 (4.0%)	
Height group (cm)			0.005
159–	148 (6.5%)	795 (5.2%)	
160–169	597 (26.1%)	3657 (24.0%)	
170–179	855 (37.4%)	6034 (39.7%)	
180–189	592 (25.9%)	3980 (26.2%)	
190+	93 (4.1%)	749 (4.9%)	
Average ALAT (mmol/l)	32.500 (19.312, 66.617)	28.500 (19.000, 53.400)	<0.001
Average Anion Gap (mmHg)	9.172 (7.145, 11.773)	8.333 (6.384, 10.189)	<0.001
Average APTT (mmHg)	44.000 (38.134, 54.000)	38.000 (34.250, 43.333)	<0.001
Average Breath Rate (g/l)	17.664 (14.146, 22.249)	16.909 (14.267, 20.013)	<0.001
Average Ca Ion (g/l)	1.143 (1.096, 1.183)	1.163 (1.127, 1.197)	<0.001
Average Calcium (mmol/l)	2.010 (1.887, 2.130)	2.020 (1.905, 2.143)	0.001
Average CK (mmol/l)	290.536 (131.438, 649.056)	319.367 (175.525, 564.979)	0.005
Average Creatinine (mmol/l)	101.345 (78.000, 148.858)	80.500 (65.000, 101.167)	<0.001
Average CRP (mmol/l)	47.342 (7.000, 126.333)	13.250 (2.600, 60.000)	<0.001
Average Diastolic Blood Pressure (mmHg)	58.327 (53.000, 64.053)	61.939 (56.519, 68.154)	<0.001
Average Glucose (mmol/l)	8.075 (7.200, 9.280)	7.944 (7.028, 8.883)	<0.001
Average Hb (mmol/l)	6.666 (6.112, 7.500)	6.909 (6.300, 7.700)	<0.001
Average Heart Rate (mmol/l)	84.231 (74.097, 96.675)	79.842 (71.300, 89.155)	<0.001
Average Inspiration Min Volume	8.656 (7.375, 10.373)	7.975 (6.950, 9.288)	<0.001
Average Leucos (mmol/l)	12.050 (9.200, 15.800)	11.775 (9.535, 14.533)	0.021
Average Magnesium (mmol/l)	0.897 (0.775, 1.060)	0.877 (0.760, 1.060)	0.001
Average O2 concentration (10^9^/l)	45.494 (40.585, 52.297)	40.286 (36.225, 44.070)	<0.001
Average O2 L/min	5.365 (4.000, 8.507)	4.400 (2.800, 5.272)	<0.001
Average O2 saturation (mmol/l)	75.053 (57.562, 88.592)	80.773 (69.538, 92.408)	<0.001
Average Systolic Blood Pressure (mmHg)	118.470 (107.857, 131.436)	126.223 (114.800, 137.886)	<0.001
Average Temperature (°C)	36.620 (35.995, 36.980)	36.713(36.299, 36.998)	<0.001
Average Thrombo (°C)	173.194 (127.856, 236.406)	190.250 (147.000, 248.000)	<0.001
Average Urine CAD (mmol/l)	91.327 (54.037, 132.036)	129.706 (94.270, 175.833)	<0.001
Average PEEP (mmHg)	8.000 (5.487, 10.110)	5.942 (5.000, 8.000)	<0.001
Average pH (l/min)	7.365 (7.317, 7.402)	7.388 (7.360, 7.413)	<0.001
Average Phosphate (mmHg)	1.110 (0.900, 1.378)	1.013 (0.851, 1.191)	<0.001
Average PO2 (mmHg)	99.188 (84.595, 120.894)	108.220 (92.150, 131.143)	<0.001
Average Potassium (mmHg)	4.154 (3.940, 4.431)	4.100 (3.907, 4.333)	<0.001
Average Prothrombin Time (cmH2O)	1.400 (1.245, 1.680)	1.256 (1.125, 1.395)	<0.001
Average ST segment (mm)	0.213 (0.123, 0.364)	0.250 (0.150, 0.433)	<0.001
Ventilated [yes]	2066 (87.0%)	12303 (76.2%)	<0.001
**APACHE II scores [day 1]**			
Total cohort	24 (18.5, 30.5)	17 (12.5, 22.5)	<0.001
Ventilated cohort	25.5 (20, 32.5)	19 (14.5, 24.5)	<0.001
Non-ventilated cohort	21 (16, 27.5)	12 (9, 16)	<0.001
**SOFA scores [day 1]**			
Total cohort	10 (7, 13)	8 (5, 11)	<0.001
Ventilated cohort	12 (9, 14)	9 (6, 11)	<0.001
Non-ventilated cohort	8 (5, 10)	4 (2, 6)	<0.001

*Numeric variables are reported with the median and interquartile range (in brackets), while categorical variables are reported with the frequency and proportion (in brackets). The resulting statistical tests (more details in the Methods section) are reported in the fourth column in the form of p-values.*

*MC: Medium Care; IC: Intensive Care; IC&MC: IC first, then MC; ALAT: Alanine transaminase; ICU: Intensive Care Unit; APTT: activated partial thromboplastin time; CK: creatine kinase; CRP: C-reactive protein; Hb: haemoglobin; urine CAD: urine output; PEEP: positive end-expiratory pressure; APACHE: Acute Physiology And Chronic Health Evaluation; SOFA: Sequential Organ Failure Assessment.*

Admissions with more than 35% missing data were excluded from the analysis. In the remaining cases, missing data for numeric variables were imputed with the median of the corresponding variable, and for categorical variables, they were imputed with the mode. Admissions and variables with dynamic features were converted into tabular representations by extracting their means.

### Ventilation Status

Patients were considered to have been ventilated if they were explicitly recorded in the database as having been ventilated, i.e., patients that have associated an item in table “processitems” indicating ‘Ventilate’. In addition, patients that did not have an explicit record in the “processitems” table of having been ventilated, but had O2 concentration or FiO2 records associated with their admission, were also classified as ventilated. This definition includes patients receiving invasive and non-invasive ventilatory support.

### Model Outcome

The outcome to be predicted in this model was the first documented episode of AF in patients admitted to ICU in sinus rhythm. As a previous history of AF is not coded in the AmsterdamUMC database, this outcome does not discriminate between new onset and pre-existing AF.

### Univariate Analysis

For univariate analysis, medians and interquartile ranges were calculated for continuous variables and frequencies and proportions for categorical variables. Differences between AF vs. non-AF patients were assessed using Kruskal-Wallis rank sum and Chi-square tests. Acute Physiology And Chronic Health Evaluation II (APACHE II) and Sequential Organ Failure Assessment (SOFA) scores were calculated upon admission to ICU.

### Multivariate Analysis

We performed multivariate statistical modelling using logistic regression (LR) to elucidate associations, in the form of odds ratios (OR), between the factors and the occurrence of AF. LR models the outcome probability or risk to be ‘1’ (positive class) as $P\left(Y=1\right)=1/\left(1+\exp\left[-\sum_{k=0}^{K}\beta_{k}X_{k}\right]\right)$, where {β_0_,…,β_*K*_} are the logarithms of the OR, which are estimated by maximum likelihood (19).

### Variable Selection

For the selection of variables, we ensured that for any pair of them that were considered clinically correlated, only one of them was included (usually the one with fewer missing values), e.g., for albumin and calcium, only calcium was selected. Subsequently, pairwise correlations between variables were calculated to verify that the variables included in the study were not highly collinear (above 0.7 using Pearson correlation). Relevant input variables were automatically selected using a sequential forward search algorithm using 3-fold cross-validation. The selection algorithm starts with a baseline model (i.e., all coefficients but the intercept set to zero, β_*k*≠0_=0), and in each step, the variable that most improves the performance on the validation set is added (20).

### Model Performance

Nested cross-validation was implemented, with the inner iterations to evaluate the variable selection, and the outer iterations to evaluate the training with the selected set of variables. Model performances were measured using the area under the receiver operator characteristic (AUC) curve. We report AUC means and confidence intervals (CI) for the full patient cohort, and ventilated and non-ventilated patients separately. Due to the class imbalance in the datasets, we also produced precision-recall curves to evaluate the three models developed. Their baselines were determined by the ratio of positives (P) and negatives (N) as y = P / (P + N) (21).

R version 3.6.3 was used for all analyses.

## Results

### Data Groups

**FIGURE 1:**
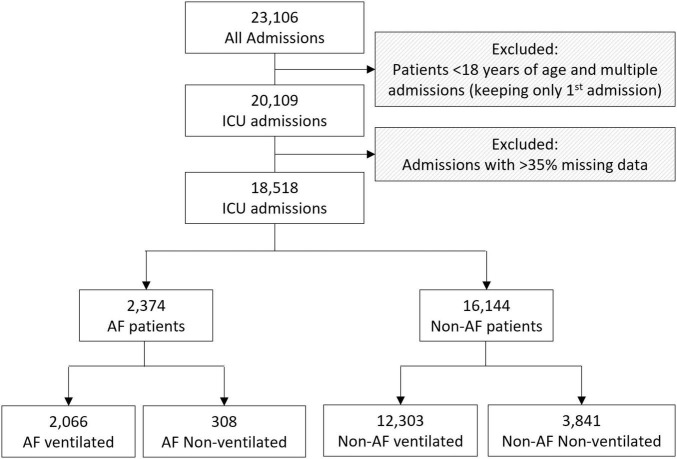
Flowchart of the cohorts analysed including the inclusion and exclusion criteria. ICU: intensive care unit; AF: Atrial Fibrillation.

From a total of 23,106 admissions extracted, patients < 18 years of age, multiple admissions and cases with > 35% missing data were excluded, resulting in 18,518 analysable cases, of which 2,374 were patients with AF patients, while 16,144 had no episodes of AF reported (Figure 1). A total of 2,066 (87%) of the patients with AF and 12,303 (76.2%) of non-AF patients required ventilation, leaving 308 (23%) AF patients and 3,841 (23.8%) non-AF patients who did not require ventilation.

Univariate statistical comparisons between AF and non-AF groups of patients are displayed in Table 1. We found statistically significant group differences (p-value < 0.05) for several vital signs, laboratory results, demographics, and the severity scoring systems. For instance, AF patients were older than 70 years (60% versus 32%) and died in ICU (23.6% versus 8%) compared to non-AF patients. We also found statistically significant differences between ventilated and non-ventilated patients (p-value < 0.001).

### Evaluation of Model Performances

**FIGURE 2:**
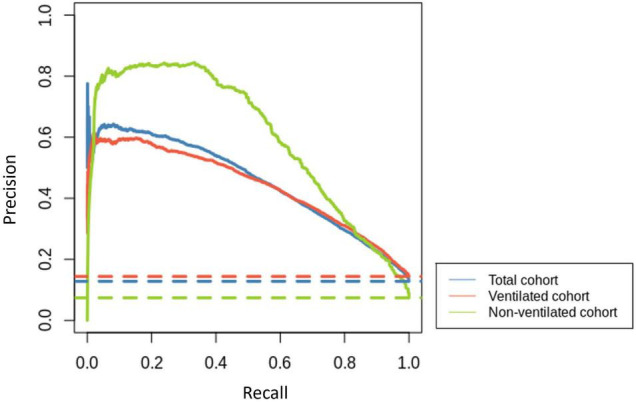
Precision-recall curves for the total cohort (blue), ventilated cohort (red) and non-ventilated cohort (green) are represented with solid lines. Their corresponding baselines are represented with dashed lines.

The developed models were able to predict the first occurrence of AF in patients admitted to critical care in sinus rhythm for all the patients in the selection group, and specifically in ventilated and non-ventilated patients, with AUC performances of 0.836 (CI: 0.833–0.838), 0.820 (CI: 0.818–0.823) and 0.912 (CI: 0.883–0.942), respectively. Additionally, the performance of disease severity scores (APACHE II and SOFA) was compared to the developed model (results in Supplementary Table 2), which as expected showed that, independently of the data cohort used, our predictive models achieved significantly better performances than severity scores developed for mortality prediction in general. The precision-recall curves for the three models, together with their baselines, are shown in Figure 2.

### Explanatory Analysis Using Logistic Regression

**FIGURE 3:**
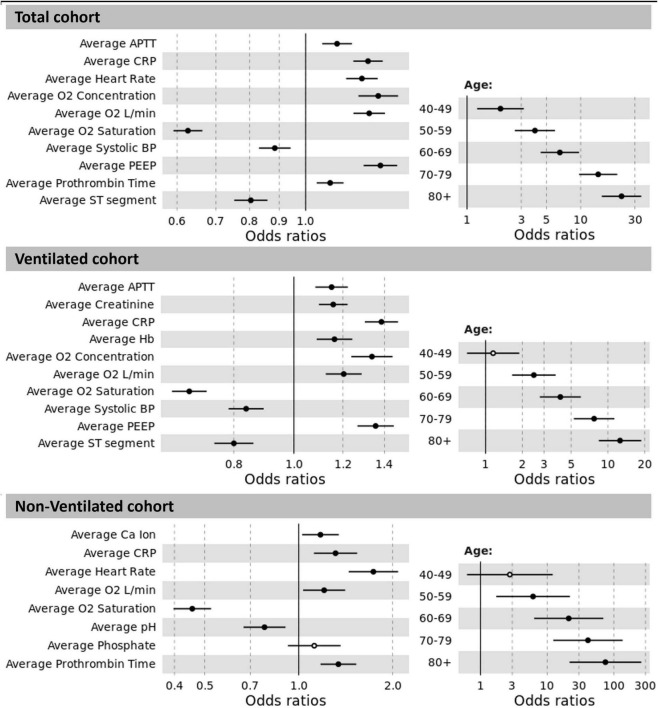
Odds ratios between significant results and prediction of AF for the total cohort, ventilated and non-ventilated cohorts. The non-significant results are displayed as hollow points. Abbreviations: APTT: activated partial thromboplastin time; CRP: C-reactive protein; O2: oxygen; BP: blood pressure; PEEP: positive end-expiratory pressure; Hb: haemoglobin; Ca: calcium.

The odds ratios (OR) for the total cohort, and the ventilated and non-ventilated patients are presented in Figure 3. We identified input variables (factors) either positively or negatively associated with the risk of developing AF in both, ventilated and non-ventilated patients, as well as in the total cohort. Factors positively associated with the development of AF in both ventilated and non-ventilated cohorts include immunoinflammatory markers such as increased average CRP (OR: total = 1.281, ventilated = 1.384, non-ventilated = 1.312) in multivariate analysis, as well as average APTT (OR: total = 1.133, ventilated = 1.150) in the ventilated cohort. Increased oxygen requirements (average O2 concentration, average O2 L/min) and reduced oxygen saturations were associated with the development of AF in both ventilated and non-ventilated patients (average O2 concentration OR: total = 1.334, ventilated = 1.336; average O2 L/min OR: total = 1.287, ventilated = 1.203, non-ventilated = 1.206). Similarly, in ventilated patients, the average PEEP (OR: ventilated = 1.355) was shown to be associated with the development of AF. Across both ventilated and non-ventilated cohorts age appears to be the single most important factor in predicting the development of AF. Patients over 80 years had the greatest risk (with OR: total = 22.909) compared to patients aged 40-49 (OR: total = 1.971). Age was even more significant in the non-ventilated cohort (with OR: non-ventilated = 74.922) in those over 80 years, compared to those aged 40-49 (OR non-ventilated = 2.757). Detailed information of the variables odd ratios and their corresponding 95% confidence intervals can be found in Table 2.

**TABLE 2:** Detailed information of the variables odd ratios (OR) and their corresponding 95% confidence intervals (CI) for each of the developed models.

Variables per cohort:	OR	95% CI
**Total cohort:**		
Age group 40–49 years	1.971	[1.229, 3.162]
Age group 50–59 years	3.960	[2.636, 5.950]
Age group 60–69 years	6.565	[4.450, 9.685]
Age group 70–79 years	14.270	[9.696, 21.002]
Age group 80+ years	22.909	[15.363, 34.163]
Average APTT (mmHg)	1.133	[1.068, 1.202]
Average CRP (mmol/l)	1.281	[1.208, 1.358]
Average Heart Rate (mmol/l)	1.250	[1.174, 1.331]
Average O2 concentration (10^9^/l)	1.334	[1.233, 1.444]
Average O2 L/min	1.287	[1.209, 1.370]
Average O2 saturation (mmol/l)	0.627	[0.592, 0.663]
Average Systolic Blood Pressure (mmHg)	0.885	[0.831, 0.942]
Average PEEP (mmHg)	1.346	[1.259, 1.438]
Average Prothrombin Time (cmH2O)	1.102	[1.045, 1.163]
Average ST segment (mm)	0.804	[0.753, 0.859]
**Ventilated:**		
Age group 40–49 years	1.153	[0.706, 1.895]
Age group 50–59 years	2.482	[1.668, 3.791]
Age group 60–69 years	4.086	[2.819, 6.110]
Age group 70–79 years	7.712	[5.345, 11.493]
Age group 80+ years	12.572	[8.549, 19.040]
Average APTT (mmHg)	1.150	[1.083, 1.222]
Average Creatinine (mmol/l)	1.157	[1.097, 1.220]
Average CRP (mmol/l)	1.384	[1.302, 1.472]
Average Hb (mmol/l)	1.163	[1.088, 1.242]
Average O2 concentration (10^9^/l)	1.336	[1.239, 1.444]
Average O2 L/min	1.203	[1.126, 1.286]
Average O2 saturation (mmol/l)	0.678	[0.636, 0.723]
Average Systolic Blood Pressure (mmHg)	0.837	[0.784, 0.894]
Average PEEP (mmHg)	1.355	[1.266, 1.449]
Average ST segment (mm)	0.800	[0.743, 0.859]
**Non-Ventilated:**		
Age group 40–49 years	2.757	[0.624, 12.943]
Age group 50–59 years	6.146	[1.876, 24.884]
Age group 60–69 years	21.090	[7.163, 80.792]
Age group 70–79 years	40.993	[13.907, 157.862]
Age group 80+ years	74.922	[24.268, 298.522]
Average Ca Ion (g/l)	1.174	[0.991, 1.324]
Average CRP (mmol/l)	1.312	[1.116, 1.537]
Average Heart Rate (mmol/l)	1.734	[1.447, 2.086]
Average O2 L/min	1.206	[1.031, 1.407]
Average O2 saturation (mmol/l)	0.456	[0.396, 0.523]
Average pH (l/min)	0.777	[0.665, 0.907]
Average Phosphate (mmHg)	1.121	[0.921, 1.359]
Average Prothrombin Time (cmH2O)	1.340	[1.177, 1.534]

## Discussion

Despite an increasing number of publications about AF in critically ill patients, its precipitants in this population are poorly understood. In this research, we identify modifiable and non-modifiable risk factors to build a logistic regression model for the prediction of a first episode of AF during admission to ICU. We analyse data from the AmsterdamUMCdb, which contains a total of 23,106 ICU admissions. Previous prediction models for AF in critical illness (16) are based on United States databases, include only septic patients (3, 16, 22) or focus on post-cardiac surgery patients (23). Although the risk of developing AF is highest in septic patients (3), prediction models for the occurrence of AF in general medical-surgical ICU populations are lacking. McMillan (24) used data from the first 8 h of ICU admission to predict subsequent AF. This approach will miss the significant proportion of critically ill patients who develop episodes of AF before ICU admission to the Emergency Department (25) or early during their ICU stay (26).

In this research, we calculate the means of time series of vital signs, laboratory results and respiratory data to build an LR model for the development of the first episode of AF in patients admitted to ICU with documented sinus rhythm. Depending on ventilation status, our model achieved very good to excellent performance measures with an AUC of 0.82 in ventilated and 0.912 in non-ventilated cohorts. The precision-recall curves (Figure 2) also support this assessment, showing that all models clearly distinguish themselves from a random classifier, indicated by their corresponding horizontal baseline. Furthermore, our models displayed good performance at predicting the small class (AF).

Since the occurrence of AF in critical illness is associated with disease severity, we used established critical care risk scores, such as APACHE II or SOFA, calculated on admission to ICU, for comparison. Our model performed significantly better in all cohorts. This may be partly explained by the limited number of variables included in the conventional risk prediction scores APACHE II and SOFA. Furthermore, the use of time series may improve model performance as dynamic changes are considered. In conclusion, while well established for mortality prediction, APACHE II and SOFA on admission are not suitable to predict AF in ICU and more specific scores are needed to identify patients at risk before the clinical onset of this arrhythmia which has repeatedly been associated with higher mortality (27, 28).

We have identified increasing age as the most important predictor of the development of AF in our analysis. Advancing age has been known as a risk factor for AF in the general population for several decades (29). Within the critical care settings, most studies investigating risk factors for AF, focus on septic patients (4, 22, 30). Despite a high level of evidence, a previous meta-analysis showed only a weak association between advanced age and AF in sepsis (22). In contrast, a scoping review (5) and a recent meta-analysis identified increasing age as the dominant risk factor in the general critical care population (13). Our model supports the role of increasing age as the principal risk factor for the occurrence of AF in critical illness: As age doubles, the risk of developing AF increases on average 10-fold. Previous work suggested that ageing in the cardiovascular system, and in particular, structural changes within the atria are major factors in the development of AF in the general population (31, 32). Bosch et al. (11) postulated that inflammation and infection can trigger accelerated cardiac structural and electrical remodelling during critical illness (11). We included CRP as a routine inflammatory marker available in the AmsterdamUMCdb in our model, and could demonstrate that higher CRP concentrations were associated with increased risk of AF.

While acute respiratory failure has been recognised as a risk factor for AF in several studies (3, 15), it remains unknown if the need for intermittent positive pressure ventilation (IPPV) is associated with a different risk profile. Clinically, the need for invasive ventilation is associated with more severe respiratory failure. Hence modelling ventilated versus non-ventilated patients separately allows prediction of AF in respiratory failure of different severity. The application of positive pressure to the airway leads to pronounced changes in intrathoracic pressure and decreases volume return to the right heart. As a consequence, left ventricular preload also decreases due to lower pulmonary venous return (33). A recent observational study in patients after cardiac surgery found a significant impact between the occurrence of AF and invasive respiratory support (34), supporting the concept that ventilated patients may exhibit a different risk profile compared to non-ventilated patients. We therefore developed three different models for risk prediction of the development of AF in a non-ventilated, a ventilated and in an undifferentiated full cohort. We also observed an impact of ventilatory settings, as higher O2 requirements and higher PEEP were associated with an increased odds ratio for the development of AF. All three models identified age as the strongest risk factor, however, in non-ventilated patients increasing age was associated with a 7-fold higher OR compared to ventilated patients. With advancing age, the severity of respiratory failure, the increased sympathomimetic activity in unsedated patients, and a lower cardiovascular tolerance to inflammation and fluid shifts, are amongst the factors which may contribute to the different weighting of risk factors depending on ventilation status.

Our study has several limitations. We performed internal cross-validation, but external validation of our model is required for generalisability across different ICU databases and before clinical implementation can be pursued. Additionally, missing data were imputed with the median/mode, which is a simple and computationally rapid approach. Alternatively, missing data imputation methods such as regression-based imputation or multiple imputation by chained equations (MICE) could be considered.

Our model was built to predict new episodes of AF in patients admitted to critical care in sinus rhythm. Insufficient information was available in the database regarding the previous medical history of paroxysmal or pre-existing AF. Thus, our model cannot predict new-onset AF, as patients with a known diagnosis of AF may also present in sinus rhythm on admission and develop episodes of AF later in their stay. Finally, in addition to the requirement for models differentiating between ventilation modes, further targeted models for individual ICU subpopulations are required, e.g., patients with sepsis, as they may display a different risk profile.

Within the AmsterdamUMC database, several variables are presented as ranges only, e.g., age, weight and height. This limits the analyses that can be performed. For example, it was impossible to calculate Body Mass Index, which is why weight and height had to be considered as separate variables in our model.

In addition to the requirement for specific models for mechanically ventilated patients, further targeted models for individual ICU subpopulations such as septic patients are required, as they may display a different risk profile.

## Conclusion

We present a logistic regression model for risk prediction of new episodes of AF in critical illness using the AmsterdamUMCdb database. Our model demonstrates very good performance in ventilated patients and excellent performance in non-ventilated patients. Further work is required to exploit the potential that different ML methods to model risk prediction for new episodes of AF in various cohorts of critically ill patients.

## Data Availability Statement

Publicly available datasets were analyzed in this study. This data can be found here: https://amsterdammedicaldatascience.nl/#amsterdamumcd.

## Ethics Statement

Ethical review and approval was not required for the study on human participants in accordance with the local legislation and institutional requirements. Written informed consent for participation was not required for this study in accordance with the national legislation and the institutional requirements.

## Author Contributions

SO-M and MP extracted and prepared the data. SO-M, IO, and MP conducted the analysis and evaluated the results. IW and BJ provided the clinical expertise and wrote the first draft of the manuscript. All authors were involved in the study design, the selection of relevant variables from the dataset, contributed to the writing, reviewing and editing, and approved the final manuscript.

## Conflict of Interest

The authors declare that the research was conducted in the absence of any commercial or financial relationships that could be construed as a potential conflict of interest.

## Publisher’s Note

All claims expressed in this article are solely those of the authors and do not necessarily represent those of their affiliated organizations, or those of the publisher, the editors and the reviewers. Any product that may be evaluated in this article, or claim that may be made by its manufacturer, is not guaranteed or endorsed by the publisher.
