# Supplementary material for: Development of a Risk Prediction Model for New Episodes of Atrial Fibrillation in Medical-Surgical Critically Ill Patients Using the AmsterdamUMCdb
Source: Front Cardiovasc Med. 2022 May 13;9:897709. doi: 10.3389/fcvm.2022.897709 (PMC9135978; doi:10.3389/fcvm.2022.897709)
Supplement: Supplementary file 1 [file Data_Sheet_1.docx]

Supplementary Material

# Dynamic features conversion into tabular representations

Dynamic features such as heart rate were converted into tabular representations (Figure S1). For this, we first calculated the mean or the recorded events per hour (in case there were multiple records in one specific hour but not as many in another hour). Then, we calculated the mean of the hourly averages (the ones that were calculated previously).


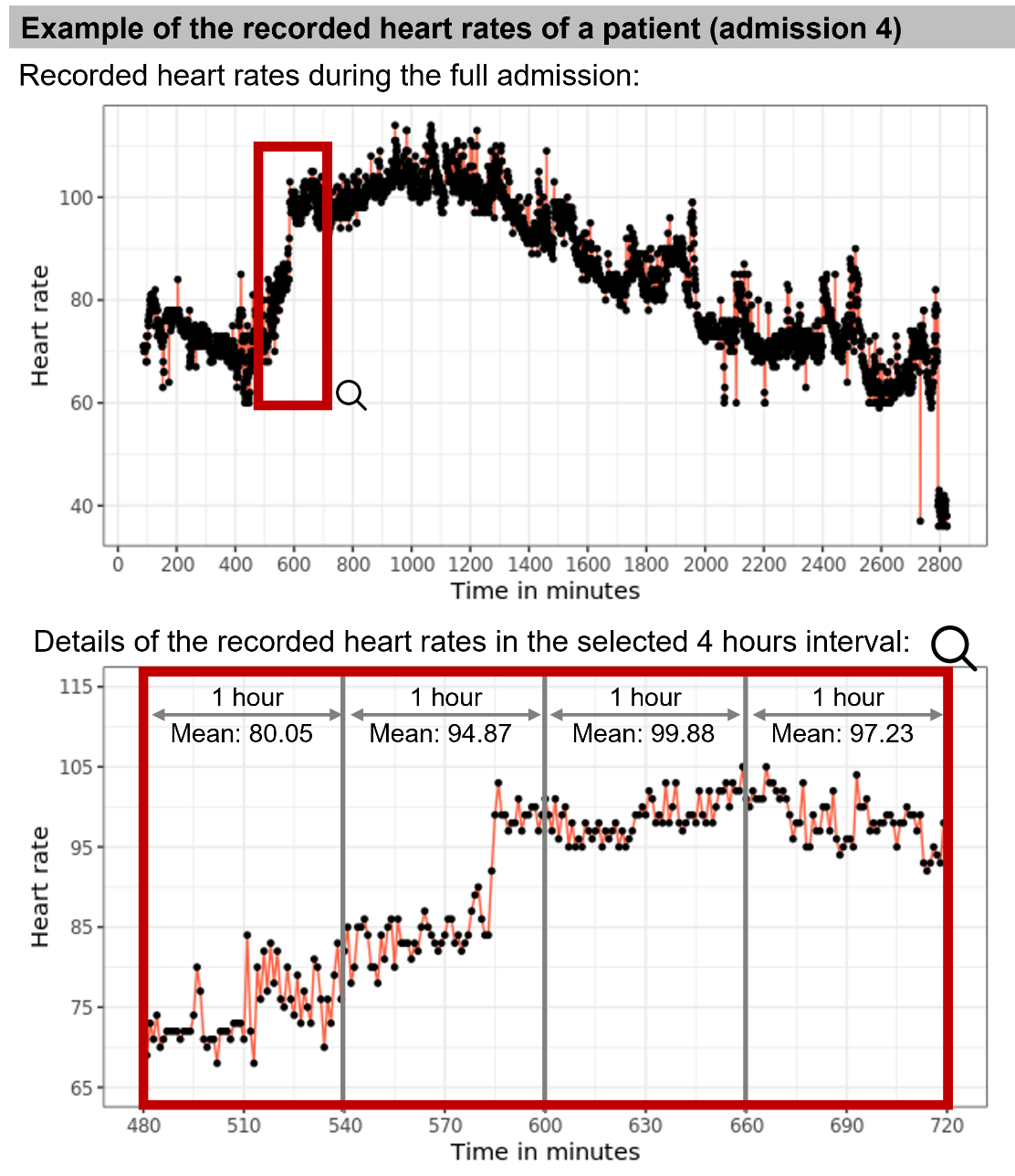


**Supplementary Figure 1.** Example of how dynamic features such as heart rate were converted into tabular representations. Firstly, the mean was calculated per hour, and then the mean of the hourly averages was calculated. Top: The recorded heart rates of one of the patients during admission. Bottom: Details of the recorded heart rates in the selected 4 hours interval (zooming in the area marked with a red rectangle on the top plot), showing how the averages per hour were calculated.

# List of variables included in the study

**Table S1.** List of variables included in the study and their level of missingness.

| Variables | **% missingness** |
| --- | --- |
| Location | 0.3 |
| Urgency | 0.0 |
| Admission year group | 0.0 |
| Gender | 0.0 |
| Age group | 0.0 |
| Weight group | 3.3 |
| Height group | 5.5 |
| Average ALAT | 22.0 |
| Average Anion Gap | 23.9 |
| Average APTT | 6.0 |
| Average Breath Rate | 6.2 |
| Average Ca Ion | 23.8 |
| Average Calcium | 12.7 |
| Average CK | 15.7 |
| Average Creatinine | 0.3 |
| Average CRP | 12.9 |
| Average Diastolic Blood Pressure | 5.8 |
| Average Glucose | 0.1 |
| Average Hb | 0.0 |
| Average Heart Rate | 0.7 |
| Average Inspiration Min Volume | 30.6 |
| Average Leucos | 3.2 |
| Average Magnesium | 8.4 |
| Average O2 concentration | 22.7 |
| Average O2 L/min | 14.9 |
| Average O2 saturation | 0.1 |
| Average Systolic Blood Pressure | 5.7 |
| Average Temperature | 1.1 |
| Average Thrombo | 0.2 |
| Average Urine CAD | 5.5 |
| Average PEEP | 26.3 |
| Average pH | 1.3 |
| Average Phosphate | 11.4 |
| Average PO2 | 1.5 |
| Average Potassium | 0.0 |
| Average Prothrombin Time | 5.7 |
| Average ST segment | 3.1 |

# Comparison with outcome prediction scores

**Table S2.** Model performance comparisons as measured using the area under the ROC curve (AUC) for the established outcome prediction scores (APACHE II and SOFA) and our novel AF prediction model. Confidence intervals are included (in brackets).

|  | APACHE II | SOFA | Our model |
| --- | --- | --- | --- |
| Total cohort | 0.746 (0.740-0.752) | 0.712 (0.700-0.724) | 0.836 (0.833-0.838) |
| Ventilated cohort | 0.719 (0.712-0.725) | 0.690 (0.684-0.697) | 0.820 (0.818-0.823) |
| Non-ventilated cohort | 0.825 (0.804-0.847) | 0.766 (0.705-0.827) | 0.912 (0.883-0.942) |
